# The Role of Ghrelin in Regulating Synaptic Function and Plasticity of Feeding-Associated Circuits

**DOI:** 10.3389/fncel.2019.00205

**Published:** 2019-05-27

**Authors:** Débora Serrenho, Sandra D. Santos, Ana Luísa Carvalho

**Affiliations:** ^1^Center for Neuroscience and Cell Biology (CNC), University of Coimbra, Coimbra, Portugal; ^2^Institute for Interdisciplinary Research (IIIUC), University of Coimbra, Coimbra, Portugal; ^3^PhD Program in Experimental Biology and Biomedicine (PDBEB), University of Coimbra, Coimbra, Portugal; ^4^Department of Life Sciences, University of Coimbra, Coimbra, Portugal

**Keywords:** ghrelin, synaptic plasticity, hypothalamus, ventral tegmental area, hippocampus, feeding

## Abstract

Synaptic plasticity of the neuronal circuits associated with feeding behavior is regulated by peripheral signals as a response to changes in the energy status of the body. These signals include glucose, free fatty acids, leptin and ghrelin and are released into circulation, being able to reach the brain. Ghrelin, a small peptide released from the stomach, is an orexigenic hormone produced in peripheral organs, and its action regulates food intake, body weight and glucose homeostasis. Behavioral studies show that ghrelin is implicated in the regulation of both hedonic and homeostatic feeding and of cognition. Ghrelin-induced synaptic plasticity has been described in neuronal circuits associated with these behaviors. In this review, we discuss the neuromodulatory mechanisms induced by ghrelin in regulating synaptic plasticity in three main neuronal circuits previously associated with feeding behaviors, namely hypothalamic (homeostatic feeding), ventral tegmental (hedonic and motivational feeding) and hippocampal (cognitive) circuits. Given the central role of ghrelin in regulating feeding behaviors, and the altered ghrelin levels associated with metabolic disorders such as obesity and anorexia, it is of paramount relevance to understand the effects of ghrelin on synaptic plasticity of neuronal circuits associated with feeding behaviors.

## Introduction

The capacity to seek and consume food is critical to survival. In nature, mammals need to optimize food searches, which requires a complex interaction of behaviors. On one hand, animals need to remember where to find food; on the other, animals may overeat to prevent future famine, which can be achieved by increasing the rewarding value of food. Nevertheless, in modern western society food is abundant, easily accessible and increasing its reward value can lead to an increase in food intake above the metabolic need. The complex interplay of behaviors associated with feeding can ultimately be prejudicial and induce metabolic disorders such as obesity and anorexia.

Behaviors associated with feeding are coordinated mainly by two inter-related neurobiological systems: the hypothalamic and mesolimbic systems. The hypothalamic system is mainly activated when energy store levels are low and drives feeding to replenish energy stores (homeostatic feeding), while the mesolimbic system, which connects the ventral tegmental area (VTA) to the striatum, is activated by pleasurable (hedonic) and incentive (motivational) aspects of food. However, all foods have a rewarding value that is influenced by hunger and food availability (Perello and Dickson, 2015), suggesting that the mesolimbic and homeostatic pathways interact tightly to control feeding. Additionally, neuronal circuits that are involved in cognitive behavior, such as cortical and hippocampal circuits, are of paramount relevance in the control of feeding as food searches require memory and learning processes.

**FIGURE 1:**
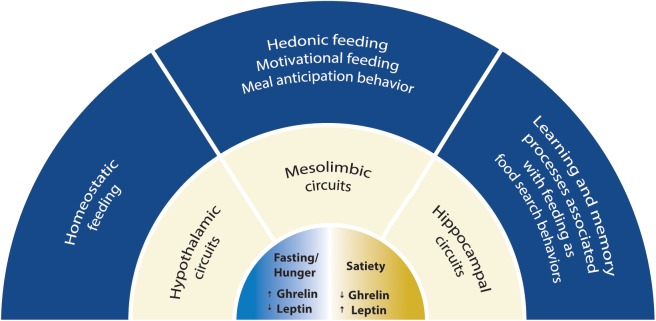
Scheme with the scope of this review. The mechanisms for synaptic plasticity modulated by ghrelin are discussed in three main neuronal circuits associated with feeding: the hypothalamic, the mesolimbic and the hippocampal circuits. Two energy states are represented: fasting/hunger and satiety. Fasting or hunger conditions are associated with high ghrelin and low leptin levels, while satiety is associated with low ghrelin and high leptin levels. Hypothalamic circuitry drives food intake depending on energy store levels (homeostatic feeding), while the mesolimbic circuitry drives consumption of food with elevated rewarding properties (hedonic and motivational feeding). Hippocampal circuitry integrates feeding with cognitive behavior suggesting that ghrelin is relevant for food searches in nature and can impact cognitive functions.

The regulation of feeding by the brain suggests that nutrients and peripheral signals released in the blood, such as glucose, free fatty acids, leptin, insulin and ghrelin can sense the energy status and reach the brain, where they modulate the activity of several neuronal circuits. Ghrelin is an orexigenic hormone affecting both energy homeostasis and higher brain functions. Ghrelin affects feeding-associated behaviors, which are accompanied by changes in synaptic strength and in the modulation of neuronal circuit function, a phenomenon termed synaptic plasticity. The effects of ghrelin on synaptic plasticity have been described in neurobiological circuits associated with feeding and cognitive behavior. In this review, we discuss the mechanisms for synaptic plasticity modulated by ghrelin in three main neuronal pathways previously associated with these behaviors: the hypothalamic, the mesolimbic and the hippocampal pathways (Figure 1). Ghrelin levels are changed in diseases linked to metabolism such as obesity and anorexia, which can lead to altered ghrelin signaling in the brain and be associated with defects on synaptic plasticity phenomena. Thus, understanding the effects of ghrelin on synaptic plasticity is critical both under physiological and pathological ghrelin signaling.

## The Ghrelin System

Ghrelin is a 28 amino acid peptide mainly released from the empty stomach (Kojima et al., 1999) but also found in other peripheral tissues such as the testis, ovary, placenta, kidney, pituitary, small intestine, pancreas, lymphocytes (reviewed in Ferrini et al., 2009; Muller et al., 2015; Mani and Zigman, 2017). Ghrelin has gained attention due to its capacity to stimulate food intake (Nakazato et al., 2001; Wren et al., 2001), to induce fat storage (Tschop et al., 2000), to prevent falls in blood glucose (Broglio et al., 2001) and to increase memory retention (Carlini et al., 2002; Diano et al., 2006). In addition, ghrelin regulates other actions such as cell proliferation, gastric acid secretion and motility (Masuda et al., 2000).

Ghrelin is present in two major forms in the blood, the acyl (octanoylated) and non-acyl forms, but only acyl-ghrelin is able to bind to the growth hormone secretagogue receptor type 1 (GSH-R1a). In fact, ghrelin is the only known peptide that undergoes *n*-octanoylation at a serine residue, enabling it to bind to GHS-R1a (Kojima et al., 1999). Ghrelin octanoylation is a unique posttranslational modification accomplished by ghrelin *O*-acyl-transferase (GOAT; Gutierrez et al., 2008; Yang et al., 2008), a member of the membrane-bound *O*-acyltransferases. GOAT utilizes dietary medium-chain fatty acids as a substrate for ghrelin acylation (Nishi et al., 2005), and acts as a nutrient sensor signaling to the brain on the presence of dietary calories (Kirchner et al., 2009). The levels of acyl-ghrelin in the blood depend on GOAT activity, since mice deficient for GOAT lack acyl-modified forms of ghrelin (Gutierrez et al., 2008). Besides *n*-octanolylated ghrelin, other acyl-ghrelin forms which include modifications with longer saturated and unsaturated fatty acyl groups have been detected (reviewed by Nishi et al., 2011).

Ghrelin acts through the GHS-R1a, which was originally described in the pituitary gland and the hypothalamus as the target of growth hormone secretagogues (Howard et al., 1996). GHS-R1a is one of the two alternative splicing forms encoded by the *Ghsr* gene (McKee et al., 1997); the other isoform, GHS-R1b, is truncated at the C-terminus, does not bind to ghrelin and possesses no signaling activity known so far. The two GHS-R isoforms can form heterodimers, which reduces the cell surface expression of GHS-R1a (Chow et al., 2012). GHS-R1a is a G-protein coupled receptor expressed in the periphery (Papotti et al., 2000) and in the brain (Guan et al., 1997; Zigman et al., 2006; Mani et al., 2014), which can signal through G protein subunit α_q/11_ and activate phosphatidylinositol-specific phospholipase C, leading to protein kinase C (PKC) activation and the regulation of ion currents. The GSH-R1a can also be coupled to activation of the phosphatidylinositol 3 (PI3)-kinase signaling cascade in different cellular systems, and lead to protein kinase A (PKA) activation (Camina, 2006). The C-terminal region of GHS-R1a is critical for ligand-induced receptor internalization, recruitment of β-arrestin_2_ and termination of GHS-R1a signaling (Evron et al., 2014). Interestingly, the GHS-R1a presents unusually high constitutive activity in the absence of the ligand (Holst et al., 2003). The physiological relevance of the GSH-R1a constitutive activity has not been fully clarified (reviewed in Mear et al., 2013), but the ligand-independent activity of the GSH-R1a is known to play a role in the control of food intake and regulation of body weight (Petersen et al., 2009; Els et al., 2012; McCoull et al., 2014; Fernandez et al., 2018), and in the acquisition of conditioned taste aversion (Li et al., 2018). Human mutations that lead to a selective loss of constitutive activity of GHS-R1a are associated with familial short stature (Pantel et al., 2006, 2009; Inoue et al., 2011). The GHS-R1a constitutive activity reduces presynaptic Ca_v_2 currents and GABA release in hypothalamic and hippocampal neurons (Lopez Soto et al., 2015; Valentina et al., 2018), and reduces the cell surface expression of Ca_v_2 channels (Mustafa et al., 2017).

Besides signaling in response to ghrelin, and in the absence of the ligand, the GSH-R1a has been shown to heterodimerize with and modulate signaling through other G-protein coupled receptors, such as dopamine D1 and D2 receptors (DR1R, DR2R), melanocortin 3 receptors and serotonin 2C receptors (Wellman and Abizaid, 2015). Recent studies provide evidence for further GSH-R1a heterodimerization with the orphan receptor G protein-coupled receptor 83 (Gpr83), which diminishes activation of GHS-R1a by ghrelin (Muller et al., 2013), and the oxytocin receptor, resulting in attenuation of oxytocin-mediated signaling (Wallace Fitzsimons et al., 2018).

In healthy humans, acute administration of ghrelin increases food intake, whether it is administered intravenously or infused (Wren et al., 2001) or subcutaneously applied (Druce et al., 2006). Similarly, in rodents, central or peripheral administration of ghrelin induces feeding/increases food intake (Tschop et al., 2000; Asakawa et al., 2001; Nakazato et al., 2001; Wren et al., 2001). Circulating ghrelin binds neurons in the vicinity of fenestrated capillaries in the arcuate nucleus of the hypothalamus (Schaeffer et al., 2013). Ghrelin levels in the blood fluctuate throughout the day in humans, rising before a meal and decreasing upon food consumption (Cummings et al., 2001), which indicates that ghrelin works as a meal initiating peptide; however, the role for ghrelin oscillations in feeding behavior has not been elucidated. The levels of circulating ghrelin are elevated in negative energy balance conditions such as in anorexia and caloric restriction, and are decreased in positive energy balance conditions such as obesity (reviewed in Muller et al., 2015). Hence, disruption of ghrelin signaling in animal models by using loss of function or gain of function studies can provide important information regarding the pathological effects of ghrelin signaling in feeding and cognitive behaviors. Interestingly, pharmacological inhibition of the ghrelin system, either by neutralizing ghrelin, inhibiting GOAT or antagonizing the ghrelin receptor caused decreased body weight or reduced food consumption (Shearman et al., 2006; Zorrilla et al., 2006; Esler et al., 2007; Barnett et al., 2010; Landgren et al., 2011), suggesting that endogenous ghrelin contributes to food intake. However, genetic mouse models of ghrelin, GHS-R1a or GOAT manipulation provided conflicting results.

Ghrelin knock-out (KO) mice did not present differences in food intake or body weight in comparison to wild-type littermates (Sun et al., 2003, 2008; Wortley et al., 2004). However, ghrelin knock-out mice were protected from the weight gain triggered by exposure to a high-fat diet at early age (Wortley et al., 2005). In addition, mice overexpressing acyl-ghrelin displayed hyperphagia, glucose intolerance, decreased glucose-stimulated insulin secretion and reduced leptin sensitivity (Bewick et al., 2009). Ghrelin did not stimulate food intake in a GHS-R KO mouse model (Sun et al., 2004; Zigman et al., 2005), showing that ghrelin acts through GHS-R to influence food consumption.

However, food intake and body weight in GHS-R KO mice fed standard chow diet were similar to their wildtype littermates (Sun et al., 2004; Zigman et al., 2005; Pfluger et al., 2008). More recently, it was showed that GSH-R1a KO rats consume less food overall at basal conditions and weigh significantly less compared with wild-type littermates throughout development (Zallar et al., 2018). Nevertheless, young GHS-R KO animals fed with a high-fat diet ate less, preferentially used fat as an energy substrate, and presented reduced body weight, and reduced adiposity and glucose levels compared to wild-type littermates (Zigman et al., 2005). Adult GSH-R KO mice did not present changes in energy expenditure or body weight under conditions of positive or negative energy balance, but showed impairment in maintaining glucose homeostasis upon caloric restriction, suggesting a function for the ghrelin receptor in modulating glucose sensing and insulin sensitivity (Sun et al., 2008). On the other hand, in aged animals fed with regular diet ablation of the GHS-R decreased body weight and reduced adiposity, as well as improved insulin sensitivity (Lin et al., 2011), similarly to what was found in young GHS-R animals fed with high-fat diet (Zigman et al., 2005). These interesting observations suggest an age-dependent role for the GHS-R1 in regulating body weight, adiposity and insulin resistance. Collectively, the ghrelin and GSH-R1a loss-of and gain-of-function studies in rodents have also demonstrated that, albeit first described as an important stimulator of food intake, ghrelin functions on energy expenditure extend to glucose tolerance and insulin sensitivity (reviewed in Mani and Zigman, 2017).

Recently, liver-expressed antimicrobial peptide 2 (LEAP2) was described as the first endogenous non-competitive antagonist of the GSH-R1a. LEAP2 is produced in the liver and small intestine, and its secretion is suppressed by fasting. LEAP2 blocks ghrelin-induced food intake, GH release and maintenance of viable glucose levels during chronic caloric restriction (Ge et al., 2018). A recent study found that in addition to antagonizing the ghrelin-induced activity of GSH-R1a, LEAP2 behaves as an inverse agonist, blocking the constitutive ligand-independent activity of the receptor (M’Kadmi et al., 2019). Since LEAP2 interacts with the GSH-R1a, and plays a role in the regulation of energy homeostasis, it is a promising therapeutic target in the treatment of metabolic diseases (reviewed in Al-Massadi et al., 2018).

## Neuromodulation of Synaptic Plasticity

Behavioral experiences such as learning or searching for food generate patterns of neuronal activity that can induce synaptic plasticity, a set of bidirectional changes in the strength of synaptic transmission (Citri and Malenka, 2008; Nicoll, 2017). During these processes, neuronal activity leads to a series of molecular and structural synaptic events, such as Ca^2+^ influx, changes in the release of neurotransmitters, alterations in receptor phosphorylation and expression at the post-synapse, changes in gene and protein expression, and modifications in the number and shape of dendritic spines. Overall, these changes are required for the expression of synaptic plasticity and learning (Citri and Malenka, 2008). In excitatory synapses, changes in the expression and biophysical properties of α-amino-3-hydroxy-5-methyl-4-isoxazolepropionic acid-type glutamate receptors (AMPARs) are major mechanisms underlying various forms of synaptic plasticity (Diering and Huganir, 2018). N-methyl-D-aspartate-type glutamate receptors (NMDAR) play a pivotal role in plasticity by allowing calcium influx to the post-synaptic cell. Calcium binds kinases including calcium/calmodulin-dependent protein kinase II (CaMKII), which can in turn phosphorylate AMPAR subunits and alter their biophysical properties and synaptic traffic (Diering and Huganir, 2018). Other kinases such as PKA and PKC can, together with CaMKII, phosphorylate AMPARs subunits and other synaptic targets, ultimately contributing to synaptic plasticity (Diering and Huganir, 2018). It is believed that synapse-specific changes in synaptic strength form the cellular basis of learning, memory and other behavioral adaptations.

The brain integrates metabolic and environmental information which is transformed into neuronal and synaptic activity, to generate behavior that promotes energy balance and survival. It is therefore not surprising that synaptic function and plasticity in brain regions that participate in different aspects of feeding behavior are modulated by the action of hormones that regulate energy homeostasis. Neuromodulators such as ghrelin and other circulating hormones act in different brain regions to activate signaling pathways that impact on synaptic function and on the induction and/or expression of synaptic plasticity mechanisms, thus affecting behavior at different levels. Interestingly, the signaling pathways downstream of GHS-R1a activation crosstalk with synaptic plasticity pathways. The ghrelin receptor activity regulates calcium intracellular levels, through activation of both the phospholipase C-PKC pathway and adenylate cyclase-PKA signaling, and leads to the activation of phosphatidylinositol 3-kinase (PI3K) (reviewed in Castaneda et al., 2010), pathways with a role in synaptic plasticity mechanisms. Through this crosstalk, ghrelin may affect synaptic function and plasticity in the hypothalamus, midbrain and hippocampus, thereby regulating homeostatic, hedonic and cognitive aspects of feeding behavior.

## Ghrelin Synaptic Signaling in the Hypothalamus

The coordinated regulation of energy intake and energy expenditure sensed by peripheral signals as a response to changes in the body energy status requires the occurrence of synaptic plasticity in the hypothalamus, which is the main region for the control of energy balance in the brain. Thus, hypothalamic synaptic plasticity is a critical process for health and survival. In the hypothalamus, the arcuate nucleus (ARC) contains the Agouti-related peptide (AgRP) or neuropeptide Y (NPY)-expressing neurons which stimulate feeding and increase body weight, and the pro-opiomelanocortin (POMC)-expressing neurons which suppress feeding in mice (Aponte et al., 2011). AgRP and POMC neurons target downstream neurons that express the melanocortin receptor 4 (MC4R). AgRP neurons suppress MC4R signaling and POMC neurons produce POMC, which is cleaved producing alpha-melanocyte stimulating hormone (α-MSH), hence increasing MC4R signaling. Thus, the balance of the firing rate of these neurons regulates feeding behaviors (reviewed in Andermann and Lowell, 2017; Sternson and Eiselt, 2017).

In fact, the opposing levels of AgRP and POMC neuronal activity in fed versus fasted animals (increased activity of AgRP neurons and reduced activity of POMC neurons in fasted animals and reduced activity of AgRP neurons and increased activity of POMC neurons in fed animals) suggest that synaptic plasticity occurs in these neurons in response to different energy status. Additionally, AgRP and POMC neurons sense peripheral signals, such as ghrelin and leptin, which modulate their activity. Ghrelin is involved in the hypothalamic regulation of feeding. The GSH-R1a is expressed in the hypothalamus (Zigman et al., 2006), and ghrelin injection intra-cerebroventricularly (i.c.v.) or in the paraventricular hypothalamic nucleus (PVN) induced feeding and the expression of c-Fos, a marker of neuronal activation, in NPY and AgRP neurons (Nakazato et al., 2001; Olszewski et al., 2003). Antagonizing NPY or AgRP signaling abolished ghrelin-induced feeding (Nakazato et al., 2001). Consistently, the orexigenic effect of ghrelin was abolished by i.c.v. co-injection of Y1 receptor antagonist, suggesting that ghrelin increases food intake in part through the activation of the NPY/Y1 pathway in the hypothalamus (Shintani et al., 2001). To test the functional significance of the action of ghrelin on AgRP neurons, GHS-R1a was specifically re-expressed in AgRP neurons of GHS-R KO mice, using a tamoxifen-inducible AgRP-CreER(T2) transgenic mouse model. GHS-R1a re-expression specifically in AgRP neurons restored the orexigenic response to administered ghrelin, suggesting that GHS-R1a-containing AgRP neurons are responsible for ghrelin’s orexigenic effects (Wang et al., 2014). Altogether, these lines of evidence suggest that ghrelin acts through hypothalamic neurons to affect energy balance and feeding behaviors.

Electrophysiological assessment of the effect of ghrelin in hypothalamic slices revealed that ghrelin decreases the activity of POMC neurons and increases the activity of AgRP neurons (Cowley et al., 2003). Activation of AgRP neurons induced the release of NPY and GABA that bind to the NPY and GABA receptors on POMC neurons (Cowley et al., 2001), leading to their hyperpolarization. Interestingly, the inhibition of GABA receptors did not alter the ghrelin-induced hyperpolarization of POMC neurons, but inhibition of both GABA and NPY receptors reversed ghrelin’s hyperpolarizing effects on POMC neurons (Cowley et al., 2003), suggesting that the effects of ghrelin in POMC hyperpolarization are induced by the upstream NPY neurons action on POMC neurons. In line with these results, a recent study showed that mice intraperitoneally (i.p.) injected with ghrelin display an increase in calcium signals in AgRP neurons *in vivo*. The POMC neurons showed the opposite response, with ghrelin injection inhibiting POMC activity (Chen et al., 2015). Altogether, these data suggest that ghrelin modulates neuronal activity in AgRP and POMC neurons. Thus, it is reasonable to hypothesize that ghrelin acts as a modulator of synaptic plasticity to regulate the activity of AgRP and POMC neurons to control feeding behaviors.

It has been hypothesized that ghrelin effects in AgRP and POMC activity can be induced by changes in the synaptic input onto these neurons. The frequency, but not the amplitude, of miniature excitatory postsynaptic currents (mEPSCs) recorded from AgRP neurons was increased in food-deprived animals (Yang et al., 2011), which show increased ghrelin circulating levels (Tschop et al., 2000). The increase in the frequency of mEPSCs onto AgRP neurons was blocked by i.c.v. injection of a GSH-R1a antagonist, suggesting that it is mediated by GSH-R1a signaling. Consistently, i.p. injection of ghrelin to fed mice (which have low levels of ghrelin) increased the frequency of mEPSC in AgRP neurons, and AgRP neuron firing (Yang et al., 2011). Additionally, ghrelin increased the frequency, but not the amplitude, of spontaneous GABAergic inhibitory postsynaptic currents onto POMC neurons *in vitro*, which was accompanied with a decrease in the activity of POMC neurons (Cowley et al., 2003). A presynaptic effect of ghrelin on both AgRP and POMC neurons suggests that ghrelin can modulate the activity of these neurons likely by targeting upstream neurons. In fact, initial work found that ghrelin-immunoreactive cell bodies are present in several hypothalamic nuclei, ghrelin is expressed in axons and is associated with dense-cored vesicles in presynaptic terminals that innervate several hypothalamic nuclei (Cowley et al., 2003). However, the detection of ghrelin in the central nervous system has been controversial, and more recent works suggest the ghrelin is produced in the brain at very low levels (reviewed in Cabral et al., 2017). It is possible that ghrelin effects on AgRP and POMC neurons are induced by peripheral ghrelin that targets GSH-R1a expressing neurons to regulate energy homeostasis.

Others mechanisms for ghrelin-induced presynaptic plasticity have been proposed. In fact, GSH-R1a is present at hypothalamic GABAergic presynaptic terminals and a recent work showed that the GHS-R1a activation elicits a strong impairment of voltage gated calcium channels Ca_V_2.1 and Ca_V_2.2 currents in hypothalamic neurons (Lopez Soto et al., 2015). Thus, ghrelin-mediated inhibition of Ca_V_2 attenuates GABA release in hypothalamic neurons, which could contribute to downstream neuronal activation through the disinhibition of postsynaptic neurons (Lopez Soto et al., 2015).

Ghrelin-evoked feeding requires signaling through energy sensors such as AMP-activated kinase (AMPK), sirtuin1 (SIRT1) or mammalian target of rapamycin (mTOR) (reviewed in Al Massadi et al., 2017). Inhibition of AMPK blocked ghrelin-mediated effects on AgRP synaptic plasticity, through a presynaptic effect dependent on Ca^2+^/calmodulin kinase kinase activation following ghrelin-triggered mobilization of Ca^2+^ from intracellular stores (Yang et al., 2011). Intriguingly, the frequency of AgRP mEPSCs was increased for hours upon ghrelin incubation, even after ghrelin removal or incubation with a GSH-R1a antagonist, and was reversed by administration of leptin and by opioid release from POMC neurons (Yang et al., 2011). This study showed that ghrelin induces long-lasting activation of AgRP neurons (a memory of the energy status), via a presynaptic AMPK-dependent mechanism that is reversed by opioids released by leptin-activated POMC neurons. Subsequent studies found that postsynaptic NMDARs in AgRP neurons are critical for fasting-induced AgRP activation and dendritic spinogenesis (Liu et al., 2012), and that inhibition of AMPK in AgRP neurons can block excitatory synaptic plasticity in AgRP neurons triggered by fasting (Kong et al., 2016). Fasting-activated AMPK phosphorylates and stimulates p21-activated kinase (PAK) signaling in AgRP neurons, which is required for fasting-stimulated synaptic plasticity (Kong et al., 2016).

SIRT1 is an energy sensor with NAD^+^-dependent deacetylase activity, which is activated in response to caloric restriction and acts through the p53 tumor suppressor. Ghrelin has been shown to trigger the SIRT1/p53 pathway, and a SIRT1 inhibitor or the deletion of p53 blunted the ghrelin-induced food intake, and impaired the effect of ghrelin on hypothalamic AMPK phosphorylation (Velasquez et al., 2011). A recent study found that mice lacking p53 in the AgRP neurons are more likely to develop diet-induced obesity, and that c-Jun N-terminal kinase (JNK) mediates the effects of AgRP neurons p53 on energy balance (Quinones et al., 2018). p53 in AgRP neurons is essential for the ability of central ghrelin to induce food intake (Quinones et al., 2018).

mTOR is a cellular sensor of changes in energy balance and nutrients, and a component of mTOR complexes (mTORC) 1 and 2. mTORC1 phosphorylates serine/threonine ribosomal protein S6 kinase 1 (S6K1), which phosphorylates and activates the ribosomal protein S6, involved in translation. mTOR is expressed in NPY/AgRP neurons (Cota et al., 2006), and ghrelin elicits an upregulation of hypothalamic mTOR activity, and an increase in the phosphorylation of the downstream targets S6K1 and S6 (Martins et al., 2012). Inhibition of hypothalamic mTOR reversed the orexigenic effect of ghrelin, and ghrelin-induced expression of NPY and AgRP (Martins et al., 2012). These observations suggest that ghrelin also promotes feeding through upregulation of the mTOR pathway in the hypothalamus.

Astrocytes modulate neuronal synaptic inputs in the central nervous system, and have been recently shown to play a role in regulating ghrelin-induced activation of AgRP neurons. *In vivo* activation of medial basal hypothalamic astrocytes reduced food intake during the early dark period (when mice usually consume more) as well as ghrelin-induced food intake. Conversely, astrocyte inactivation enhanced and prolonged ghrelin-inducing feeding (Yang et al., 2015). These effects occur by a mechanism mediated by adenosine, which is released by astrocytes and negatively regulates synaptic transmission in AgRP neurons through activation of adenosine A1 receptors (Yang et al., 2015).

**FIGURE 2:**
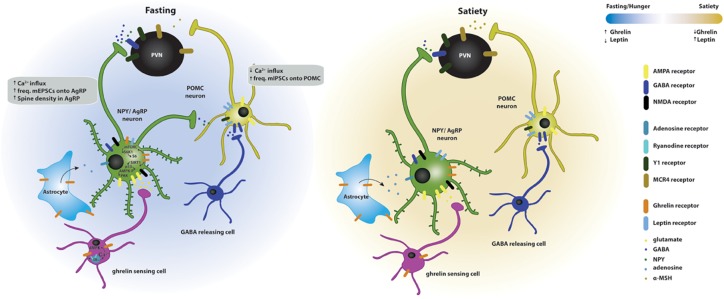
Effects of ghrelin on synaptic plasticity of hypothalamic circuits associated with feeding. Two energy states are represented: fasting/hunger and satiety. In the fasted state, AgRP neuronal activity and dendritic spine density are increased, while POMC neuronal activity is decreased. The AgRP neurons release NPY and GABA and the POMC neurons release POMC, which is subsequently cleaved to produce α-MSH, and targets the melanocortin receptors in the paraventricular nucleus (PVN MCR4) to regulate feeding responses. The effects of fasting on hypothalamic synaptic plasticity can be mediated directly by the activation of the GSH-R1a in AgRP neurons or indirectly by the upstream regulation of AgRP and POMC neurons. Upstream neurons include ghrelin-sensing cells that release glutamate on AgRP neurons through a mechanism dependent on the release of calcium from internal stores and subsequent activation of AMPK, therefore increasing the excitatory synaptic input onto these neurons. Simultaneously, ghrelin leads to the activation of NMDA receptors, of the SIRT1 → p53 → AMPK → PAK and of the mTORC → S6K1 → S6 pathways, which drive state-dependent excitatory synaptic plasticity in AgRP neurons. In parallel, a population of neurons release GABA onto the POMC neurons therefore increasing the inhibitory tone on these neurons. In the fed state, the balance of excitatory versus inhibitory synaptic inputs in the AgRP and POMC neurons is reversed, increasing the anorexigenic melanocortin tone. Additionally, astrocytes express GSH-R1a and its activation promotes the release of adenosine that will bind to the adenosine A1 receptors expressed in the AgRP neurons, thus, decreasing their excitation threshold.

Overall, ghrelin promotes feeding by acting on hypothalamic circuits (Figure 2); the mechanisms that coordinate ghrelin’s orexigenic action depend on ghrelin-induced synaptic plasticity and are starting to emerge, but need to be further clarified.

## Regulation of Synaptic Function in the VTA by Ghrelin

The mesolimbic system activation increases the motivation for food-seeking and for consumption behaviors favoring high-sucrose or high-fat over chow diets. Disruptions of the mesolimbic system are associated with addictive behaviors such as overfeeding of palatable food or drug consumption. These behaviors are accompanied by plasticity phenomena occurring on mesolimbic pathways. For instance, exposure to drugs of abuse induces synaptic plasticity in the VTA (Luscher and Malenka, 2011). Thus, plasticity of mesolimbic pathways is of particular interest in the study of behaviors associated with motivational, hedonic consumption and with increased feeding. Ghrelin increases the incentive value for natural and chemical rewards, via activation of GHS-R1a (reviewed in Perello and Dickson, 2015), which is expressed in the VTA (Guan et al., 1997; Abizaid et al., 2006; Zigman et al., 2006; Perello and Dickson, 2015). Ghrelin recruits subsets of dopamine and GABA neurons of different VTA subnuclei (Cornejo et al., 2018). The mesolimbic pathway connects the VTA containing dopaminergic neurons projecting mainly to the nucleus accumbens (NAc) in the ventral striatum but also sending projections to the hypothalamus, prefrontal cortex and the hippocampus. The VTA also receives projections from these regions and from cholinergic neurons of the laterodorsal tegmental area (LTDg). In the scope of this review, we will focus mainly in the VTA-NAc projection, as this is one of the most studied pathways in the context of ghrelin effects on feeding.

In humans, functional MRI studies showed that peripheral ghrelin altered the response of the ventral striatum to visual food cues, suggesting that ghrelin can act through the mesolimbic system to affect feeding behavior (Malik et al., 2008). In rodents, intra-VTA ghrelin injections increased food intake (Naleid et al., 2005; Abizaid et al., 2006), in particular of rewarding food (Egecioglu et al., 2010; Skibicka et al., 2012). Knock-out mice for the GHS-R1a, rats peripherally treated with a GHS-R1a antagonist, and VTA-lesioned rats showed suppressed intake of rewarding foods (Egecioglu et al., 2010). Consistently, ghrelin peripheral injection increased the motivation to consume high-fat diet when administered to *ad libitum*-fed mice, but both wild-type mice treated with a GSH-R1a antagonist and GSH-R KO mice failed to show the preference for a high-fat diet normally observed in animals under calorie restriction (Perello et al., 2010). This line of evidence suggests that ghrelin increases the motivation for calorie dense food through its actions on the VTA.

Interestingly, the reward value of food can compensate for defective feeding in mice in which AgRP neurons were selectively ablated (Denis et al., 2015). Ghrelin i.p. injection did not trigger chow intake in mice lacking AgRP neurons, but it significantly increased consumption in animals on a high fat and high sugar diet (Denis et al., 2015). Intra-VTA ghrelin administration was sufficient to trigger an increase in high fat and high sugar (but not chow) diet intake in AgRP-ablated mice, supporting the hypothesis that VTA ghrelin is orexigenic in mice fed a palatable diet in the absence of AgRP neuronal activity (Denis et al., 2015). Long-term exposure to high fat and high sugar diet results in ghrelin resistance in AgRP neurons (Briggs et al., 2010; reviewed in Zigman et al., 2016). In these circumstances, feeding behavior may result from the engaging of AgRP-independent but ghrelin-dependent neural circuits modulated by food palatability and dopamine signaling.

Natural and artificial rewards increase the activity of the VTA dopaminergic neurons, triggering the release of dopamine in the NAc and an increase in locomotor activity. Likewise, central or intra-VTA administration of ghrelin induced locomotor stimulation and dopamine-overflow in the NAc, whereas intra-VTA administration of a GHS-R1A antagonist suppressed this effect (Jerlhag et al., 2006, 2007). Moreover, peripheral ghrelin injections increased dopamine turnover in the NAc (Abizaid et al., 2006; Jerlhag et al., 2011), which was blocked by intra-VTA administration of a GHS-R1a antagonist (Jerlhag et al., 2011), indicating that activation of the mesolimbic dopamine system by ghrelin requires GHS-R1a signaling in the VTA. The overflow of dopamine is thought to increase the motivation to seek and consume food. Mechanistically, ghrelin was found to increase the frequency of action potentials (APs) in VTA dopaminergic neurons in brain slices, and peripheral ghrelin injection increased the number of excitatory synapses onto VTA dopaminergic neurons, while decreasing the number of inhibitory synapses. Accordingly, the frequency of mEPSCs recorded from dopaminergic neurons in VTA slices from mice peripherally injected with ghrelin was increased, whereas the frequency of miniature inhibitory postsynaptic currents (mIPSCs) was decreased (Abizaid et al., 2006). These results suggest that ghrelin induces synaptic rearrangements on the VTA dopaminergic neurons that result in an increase on the number of excitatory synapses.

The activity of VTA dopaminergic neurons is modulated by various afferents to the VTA, including glutamate-, opioids- and orexin-releasing neurons, suggesting that ghrelin-induced increase in VTA dopaminergic activity could be indirectly mediated by the activation of these pathways. Ghrelin increased the rewarding value of high-fat diet in an orexin-dependent manner, since both orexin-deficient mice and wild-type mice treated with an orexin receptor antagonist failed to show preference to rewarding food diet induced by ghrelin (Perello et al., 2010; Cone et al., 2014). However, intra-VTA administration of an NMDAR antagonist attenuated ghrelin-induced locomotor stimulation in mice, which was not affected by an orexin A receptor antagonist or peripheral injection of an opioid receptor antagonist (Jerlhag et al., 2011). Moreover, ghrelin did not affect the frequency of APs in VTA dopaminergic neurons in the absence of excitatory synaptic input, whereas it increased the frequency of APs in VTA dopaminergic neurons when ionotropic GABA_A_ receptors were blocked (Abizaid et al., 2006). Collectively, these observations suggest that the ghrelin-mediated increase in the frequency of APs in dopaminergic VTA neurons requires excitatory inputs to these neurons. In fact, blockade of NMDAR in the VTA reduced both food- and ghrelin-induced NAc dopamine release and abolished ghrelin-induced locomotor stimulation (Abizaid et al., 2006; Jerlhag et al., 2011). The ghrelin effects on the reward pathway may also be mediated by cholinergic afferents from the LTDg to the VTA, which contribute to the regulation of motivated behaviors. Thus, another possibility is that the activation of nicotinic acetylcholine receptors expressed in cholinergic presynaptic neurons is involved in the ghrelin-induced rewards. This is supported by findings showing that the GSH-R1a is expressed in the cholinergic afferents coming from the LTDg and that peripheral injection of an unselective nicotinic antagonist blocks ghrelin-induced effects (Jerlhag et al., 2006). The cannabinoid system stimulates food intake and impacts on body weight, partially through modulation of the orexigenic effect of ghrelin. Rimonabant, an antagonist of the cannabinoid receptor type 1 (CB1), blocked the orexigenic effect of ghrelin, and ghrelin could not stimulate feeding in CB1 KO mice (Kola et al., 2008).

**FIGURE 3:**
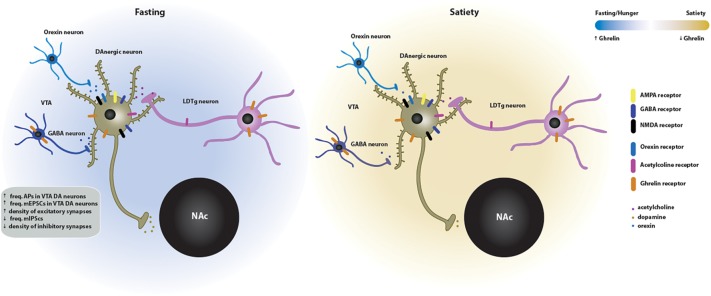
Effects of ghrelin on synaptic plasticity in the VTA → NAc circuit of the mesolimbic system. Two energy states are represented: fasting/hunger and satiety. In the fasted state, ghrelin increases the neuronal activity and the density of excitatory synapses of dopaminergic neurons in the VTA. This increase in VTA dopaminergic activity triggers the release of dopamine onto the NAc. The ghrelin effects on the VTA synaptic plasticity may be directly mediated since the GSH-R1a is expressed in the VTA DA and GABA neurons. Indirect activation of the VTA-NAc pathway by ghrelin is mediated by orexin, which is released by upstream orexigenic neurons, and by cholinergic afferents from the laterodorsal tegmental area (LDTg), which contain both GSH-R1a and nicotinic acetylcholine receptors in cholinergic presynaptic neurons. GABAergic transmission is also involved in the ghrelin-induced effects on neuronal activity. In the fed state, the VTA neuronal activity and, subsequently, dopamine release on the NAc return to basal levels.

Taken together, these data suggest that ghrelin effects on synaptic function may be directly mediated by the GSH-R1a or indirectly by the activation of afferents from other brain regions. Moreover, neurotransmitters including acetylcholine and glutamate are required for ghrelin-induced reinforcement. These pathways in the VTA (Figure 3), that appear to be sensitive to cholinergic and glutamatergic input, may serve as a novel pharmacological target for treatment of ghrelin-induced addictive behaviors. Other studies have shown that ghrelin increases (whereas antagonists of the GHS-R1a antagonists decrease) the motivation to consume alcohol, and that ghrelin signaling is required for the rewarding properties of addictive drugs (reviewed in Panagopoulos and Ralevski, 2014). Modulation of the effect of ghrelin in the mesolimbic system may offer a potent therapeutic strategy to target the ghrelin-induced increase in the reward value of food and drugs of abuse. The recent discovery of the endogenous GHS-R1a antagonist LEAP2 (Ge et al., 2018) opens the way to testing whether modulating its levels in the mesolimbic system can be of therapeutic value.

## Synaptic Modulation by Ghrelin in the Hippocampus

Expression of the GHS-R1a in the hippocampus suggests that ghrelin is involved in hippocampal-dependent functions, and that this hormone may be a link between metabolism and cognition. In fact, intracerebroventricular (Carlini et al., 2002; Diano et al., 2006), intra-hippocampal (Carlini et al., 2004) or peripheral (Diano et al., 2006) injections of ghrelin increased hippocampal-dependent memory retention. In addition, ghrelin-null mice showed impairments in recognition memory (Diano et al., 2006), and GSH-R KO animals displayed spatial and contextual memory impairments (Davis et al., 2011; Albarran-Zeckler et al., 2012), suggesting that endogenous ghrelin signaling modulates cognitive behavior. The hippocampus is an anatomically defined structure in the brain composed of the Cornu Ammonis regions [I (CA1), II (CA2) and III (CA3)] and the dentate gyrus (DG). Synaptic plasticity paradigms of long-term potentiation (LTP) and long-term depression (LTD) are cellular correlates for learning and memory that have been described in detail in this brain region (reviewed in Nicoll, 2017).

Ghrelin receptor activation enhanced LTP at the Schaffer collateral-CA1 synapse (Diano et al., 2006; Ribeiro et al., 2014) in hippocampal slices, and led to the synaptic insertion of AMPAR through a mechanism dependent on PI3K, PKA and PKC activation (Ribeiro et al., 2014). *In vivo* a single infusion of ghrelin induced long lasting potentiation of synaptic transmission in the DG, and prevented decline in LTP (Chen et al., 2011). Ghrelin-induced potentiation of synaptic transmission in the DG involved both postsynaptic and presynaptic mechanisms, did not require NMDAR activation, and was dependent on activation of the PI3K pathway (Chen et al., 2011). Consistently, enhancement of spatial memory by hippocampal infusion of ghrelin was prevented by the blockade of PI3K (Chen et al., 2011). Synaptic plasticity is associated with changes at the microstructural level, specifically at dendritic spines. Ghrelin peripheral injections increased the density of dendritic spines in the hippocampus (Diano et al., 2006), and ghrelin incubation of hippocampal organotypic slices increased filamentous-actin, the main cytoskeleton component of dendritic spines (Berrout and Isokawa, 2012). Collectively, these findings suggest that the effects of ghrelin on synaptic and structural plasticity regulate hippocampal dependent-functions.

In the hippocampus, GSH-R1a-DR1R heteromers are involved in the regulation of hippocampal synaptic plasticity (Kern et al., 2015). In fact, activation of DR1Rs increased the intracellular levels of calcium, which was not observed in hippocampal neurons obtained from GSH-R KO mice. GHS-R1a-DR1R heteromers interact with Gα_q_, and activation by a DR1R agonist led to phospholipase C activity in wildtype but not in GSH-R KO neurons, at the expense of canonical Gα_s_ cAMP signaling downstream of DR1R (Kern et al., 2015). DR1R agonist-induced activation in hippocampal organotypical slices increased the phosphorylation of CaMKII and AMPAR subunits at serine residues relevant for plasticity, in a manner dependent on GHS-R1a. Consistently, DR1R agonist-induced activation increased the surface expression of AMPAR in wildtype but not GHS-R KO hippocampal neurons. In agreement with this molecular mechanism, DR1R activation modulated hippocampal-dependent behavior in a GHS-R1a-dependent manner (Kern et al., 2015).

Additionally, similarly to what was described in the hypothalamus (Lopez Soto et al., 2015), Ca_V_2 voltage-gated calcium channels were found to be inhibited by GSH-R1a activity in the hippocampus. In fact, agonist-independent GHS-R1a activity inhibited Ca_V_2.2 channels, which decreased GABA release in hippocampal cultures, suggesting a potential physiological role for GSH-R1a constitutive signaling in regulating synaptic transmission in the hippocampus (Martinez Damonte et al., 2018).

The hippocampus and the hypothalamus communicate to regulate high-order aspects of feeding. Although ghrelin-induced feeding has been associated with cognitive changes at the hippocampus, it has been proposed that ghrelin can act in the same circuitry in the opposite direction thus suggesting that ghrelin-induced cognitive effects can influence feeding (reviewed in Hsu et al., 2016). Injection of ghrelin in the ventral subregion of the hippocampus (VH, but not in the dorsal hippocampus) increased feeding primarily by increasing meal frequency and spontaneous meal initiation in *ad libitum* fed rats (Kanoski et al., 2013). The ghrelin-induced feeding was blocked by co-administration of a PI3K inhibitor suggesting that PI3K-Akt signaling in the VH is required for the hippocampal ghrelin effects on food intake (Kanoski et al., 2013). Interestingly, a recent study showed that ghrelin signaling in the VH is important for conditioned feeding behavior (Hsu et al., 2015). Ghrelin can stimulate meal-entrained conditioned appetite by acting in the VH, which GHS-R1a-expressing CA1 neurons provide input to neurons of the lateral hypothalamic area (LHA). Lesions in LHA block the hyperphagic response induced the GHS-R1a activation in VH neurons (Hsu et al., 2015), suggesting that this pathways is required for ghrelin-induced effects on entrained appetite. Furthermore, activation of downstream orexin-1 receptors was required for VH ghrelin-mediated hyperphagia (Hsu et al., 2015). These findings reveal a novel neurobiological circuitry regulating appetite through hippocampal GSH-R1a signaling. Social aspects of feeding behavior are also regulated by ghrelin action in the hippocampus. Ghrelin targeted to the VH CA1 region enhanced social transmission of food preference in rats, and this learning behavior was eliminated following knockdown of GHS-R1a in the VH (Hsu et al., 2018).

**FIGURE 4:**
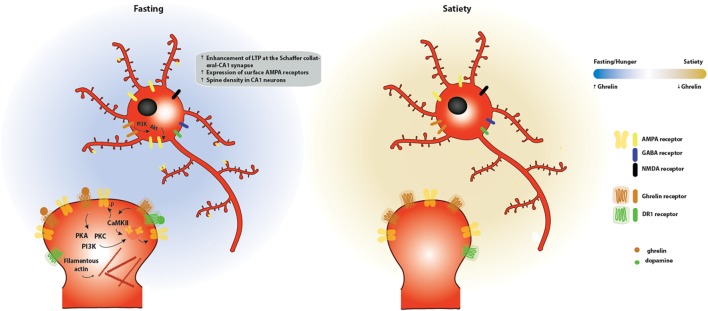
Effects of ghrelin on synaptic plasticity in the hippocampus. Two energy states are represented: fasting/hunger and satiety. During fasting or hunger, ghrelin increases the expression of LTP at the Schaffer collateral-CA1 synapse promoting the insertion of AMPA receptors through a mechanism dependent on PI3K, PKA and PKC activation. These functional changes are accompanied by an increase in the density of dendritic spines, as a result of an increase in the levels of filamentous actin. One potential mechanism for the effects of ghrelin on hippocampal plasticity is through activation of GSH-R1a-DR1R heteromers by dopamine, which promotes an increase in the phosphorylation of CaMKII and of AMPA receptor subunits at serine residues relevant for plasticity, leading to the synaptic incorporation of AMPA receptors in a GHS-R1a-dependent manner.

Taken together, these different lines of evidence support the hypothesis that ghrelin affects hippocampal-dependent functions (Figure 4). Nevertheless, a link between synaptic plasticity and ghrelin-dependent behavior is missing. Further manipulation of ghrelin signaling in the aforementioned circuits would be important to evaluate specific roles of ghrelin in hippocampal-dependent cognitive and feeding behavior. Understanding these ghrelin signaling pathways could have therapeutic value in cognitive deficits linked to metabolic disorders.

## Concluding Remarks

Ghrelin signaling is strongly linked to alterations in synaptic function in circuits regulating homeostatic, hedonic and cognitive aspects of feeding behavior. The orexigenic effects of ghrelin depend on its actions in the hypothalamus, where it modulated neuronal activity in AgRP and POMC neurons. The signaling downstream of the ghrelin receptor activation in the hypothalamus is complex, and may involve parallel pathways that include SIRT1/p53, AMPK and the mTOR pathways, which are crucial for the orexigenic action of ghrelin. It is now clear that besides its function in homeostatic feeding behavior, ghrelin plays a major role in hedonic and motivational aspects of feeding, through activation of dopaminergic signaling in the mesolimbic system. Additionally, the observed effects of ghrelin on cognitive behavior likely result from its actions in the hippocampus. The knowledge of the neuronal circuits impacted by ghrelin and of the molecular underpinnings of ghrelin’s action in these systems has progressed in recent years, but further research is required to understand in an integrated manner how ghrelin regulates the multifactorial aspects of feeding behavior. In particular, when considering the ghrelin system as a therapeutic target in metabolic disorders it is important to ponder how interfering with ghrelin signaling may affect aspects of cognition. On the other hand, the dual role of ghrelin in metabolism and cognition poses an opportunity for targeting the ghrelin system in neurodegenerative disorders such as Alzheimer’s and Parkinson’s disease (Shi et al., 2017). In modern societies plagued by an epidemics of overeating, weight gain and associated disorders, this understanding may be crucial to design better therapeutic interventions for patients with metabolic diseases.

## Author Contributions

DS, AC, and SS wrote the manuscript. DS made illustrations.

## Conflict of Interest Statement

The authors declare that the research was conducted in the absence of any commercial or financial relationships that could be construed as a potential conflict of interest.
